# Fuzi Attenuates Diabetic Neuropathy in Rats and Protects Schwann Cells from Apoptosis Induced by High Glucose

**DOI:** 10.1371/journal.pone.0086539

**Published:** 2014-01-23

**Authors:** Jing Han, Peng Tan, Zhiyong Li, Yan Wu, Chun Li, Yong Wang, Beibei Wang, Shuang Zhao, Yonggang Liu

**Affiliations:** 1 Beijing University of Chinese Medicine, Beijing, China; 2 Minzu University of China, Beijing, China; UAE University, Faculty of Medicine & Health Sciences, United Arab Emirates

## Abstract

Radix aconite lateralis preparata (Fuzi), a folk medicine, has long been used for the treatment of diabetes and paralysis in China. We examined the effect of Fuzi alone on diabetic rats and Schwann cells in high glucose and the components responsible for its activity. The major constituents of FZE were identified by HPLC-MS/MS data. Male Sprague Dawley rats (n = 36) were randomly divided into control, diabetic, FZE 1.75 g/kg, FZE 3.50 g/kg, FZE 7.00 g/kg, and methylcobalamin groups. After two weeks treatment, nerve conduction velocity and paw withdrawal latency were measured. In vitro, the Schwann cells were grouped according to exposure: normal glucose (NG), normal glucose plus mannitol (NG+M), high glucose (HG), and HG plus different concentrations of FZE (0.1 µg/ml, 1.0 µg/ml, and 10.0 µg/ml). Oxygen free radicals and apoptosis were evaluated through DCFH_2_DA, DHE and annexin-PE/7-AAD assay, respectively. Apoptosis factors (Bax, Bcl-2, CytoC, caspase-3, and caspase-9) were analyzed using immunofluorescence. Nine alkaloids were identified. The results from animal model showed that FZE was effective in accelerating nerve conduction velocity and shortening paw withdrawal latency in diabetic rats. And in vitro, FZE was also found to protect Schwann cells against high glucose injury. FZE could significantly decrease the apoptotic ratio, superoxide anion and peroxide level. Furthermore, the apoptosis factors, including Bax, Bcl-2, CytoC, caspase-3, and caspase-9 were ameliorated in FZE treated groups. The HPLC-MS^n^ method is simple and suitable for the identification of alkaloids in Fuzi. FZE has a protective effect in diabetic neuropathic rats, which is probably achieved by the antiapoptotic effect of FZE on Schwann cells. Apoptosis factor data imply that FZE protected Schwann cells through the mitochondria pathway. Alkaloids are major components contributing to the protective effect.

## Introduction

Diabetes mellitus is one of the most serious problems across the World. 347 million people worldwide have suffered from diabetes [www.who.org]. Over time, diabetes can damage the heart, blood vessels, eyes, kidneys, and nerves. WHO projects that diabetes will be the 7^th^ leading cause of death in 2030 [www.who.org]. The prevalence of neuropathy in diabetic patients is about 30%, whereas up to 50% of patients will certainly develop neuropathy during their disease [1]. Early disorders of nerve function include slowing in nerve conduction velocity and abnormal thermal perception followed by axonal degeneration, paranodal demyelination and loss of myelinated fibers [2]–[3]. Diabetic peripheral neuropathy (DPN) has been a major cause of morbidity and mortality, and is a leading risk for foot ulceration and eventual limb amputation.

Glycemic control is the only proven disease-modifying treatment which can prevent the progression of DPN, but its role in the regression of established DPN is controversial. There is a wide range of drugs that can help patients with DPN; however, the long-term efficacy and safety of these agents are not well established [4]. So the exploration for new drugs has been a major trend.

Aconite (Fuzi) is a well-known traditional Chinese medicinal herb, which is widely used clinically for treatment of acute pancreatitis, hypertension [5], heart failure [6], arrhythmia [7], neuropathic pain [8] and rheumatoid arthritis [9]. Originally in the Eastern Han Dynasty of China (24–220 AD), Fuzi was recorded by ‘Shennong Materia Medica’ (Shennong BenCaoJing), the earliest Pharmacopeia of China [10]. Latterly, it was described for its medicinal effect against diabetes by ‘San Franciscans overall record’ (Shengji Zonglu), paralysis (Chinese: Weizheng) and pain by ‘Treatise on Febrile Diseases’ (Shang HanLun) [11]–[12]. Modern clinical studies also have evidenced that Fuzi in combination with other herbs have treatment effect on the patients with DPN or pain, and in those studies, the EMG and symptoms of patients were improved [13]–[14]. In animal studies, it was reported that Fuzi could ameliorate pain in rats or mice. After the sciatic nerve was injured, it shortened mechanical withdrawal threshold, and increased thermal withdrawal duration [15]. In normal mice, Fuzi decreased the times of the acetic acid-induced writhing [16]–[17]. The chemical composition is mainly diterpenoid alkaloids, including aconitine, mesaconitine and hypaconitine, and so on [18]–[19]. The traditional use is not sufficient to validate Fuzi as an effective and safe drug for DPN. So, it is important and necessary to confirm its efficacy and the effective components.

In the present study, the protective effects of FZE on diabetic neuropathy were assessed in STZ-induced diabetic rats. Moreover, in order to clarify the underlying molecular mechanism, we evaluated the impact of FZE on the oxidative stress, apoptosis and related apoptotic factors in cultured Schwann cells. Besides, the major constituents of FZE were identified using HPLC-MS-MS.

## Materials and Methods

### Animals

Healthy male Sprague-Dawley rats (300–350 g) were supplied by Vital River Laboratory Animal Technology Co. Ltd. (Beijing, China, Certificate No. SCXK (Beijing) 2006–0001). The animals were housed in stainless-steel cages in a room with controlled temperature (25±1°C) and humidity (65±5%) and a 12-h light/dark cycle. The animals were fed with standard diet and had free access to water. All procedures involving animals and their care were carried out according to the governmental guidelines on animal experimentation, National Institutes of Health “Principles of Laboratory Animal Care”. All the experimental protocols were approved by the Institutional Animal Ethics Committee of Beijing University of Traditional Chinese Medicine, Beijing, China.

### Sample Preparation

For HPLC-DAD-MS^n^ analysis of Fuzi extract, Fuzi was pulverized in a mechanical grinder. The powder was extracted one time with 0.5 mol/l hydrochloric acid solution under reflux for 200 minutes. After filtration and combination of the filtrates, they were concentrated to 1.60 g/ml. The solution was filtered through 0.45 µM membranes prior to use, and a 10 µl aliquot was injected for analysis.

### Plant Material

The aerial part of Fuzi was collected from Sichuan province, China, in July 2011. The herb was authenticated by Dr. Peng Tan (School of Chinese Medicine, Beijing University of Chinese Medicine, Beijing). A voucher specimen was stored at School of Chinese Meteria Medica, Beijing University of Chinese Medicine, Beijing, China (No. 20110728).

### HPLC-MS^n^ Analysis

HPLC-MS^n^ analysis was performed on an Agilent series 1100 HPLC instrument (Agilent, Waldbronn, Germany) coupled with an Agilent 1100 MSD XCT/plus ion-trap mass spectrometer (Agilent, Waldbronn, Germany) via an electrospray ionization (ESI) interface. The HPLC instrument was equipped with an auto sampler, a quaternary pump, and a column compartment. Samples were separated on a Zorbax Extend-C18 column (150 mm×4.6 mm I.D., 5 µm). The mobile phase consisted of acetonitrile (A) and water containing 0.05% (v/v) ammonia water (B). A gradient program was used as follows: 0 min, 5∶95 (A: B, v/v); 10 min, 18∶82; 45 min, 32∶68; 60 min, 45∶55. 15 min post-run time was set to fully equilibrate the column. The flow rate was 0.8 ml/min. The column temperature was 25°C. The sample injection volume was 10 µl. The HPLC eluent was introduced into ESI source of mass spectrometer in a post column splitting ratio of 5∶1. For MS detection, high purity nitrogen (N_2_) was used as the nebulizing gas, and ultra-high pure helium (He) as the collision gas. Positive ion polarity modes were used for compound ionization. The ESI source parameters were optimized by injecting a 7 µl/min flow of aconitine to obtain maximum intensities of ions. The optimized parameters in the positive ion mode were as follows: source voltage, 4.4 kV; sheath gas (N_2_), 38 arbitrary units; auxiliary gas (N_2_), 10 units; capillary temperature, 330°C; capillary voltage, −28 V; tube lens offset voltage, −20 V. In the positive ESI ion mode, the capillary voltage was 15 V, and the tube lens offset voltage was 37V. For full scan MS analysis, spectra were recorded in the range of m/z 100–1000. The data-dependent program was set so that the two most abundant ions in each scan were selected and subjected to tandem mass spectrometry (MS^n^, n = 3). The isolation width of precursor ions was 2.0 Th.

### Reagents and Antibodies

Streptozotocin and mannitol were purchased from Sigma Chemical Co. (St. Louis, MO, USA). Carboxy-H_2_DCFDA (5-(and-6)-carboxy-2′,7′-dichlorodihydrofluorescein diacetate) and DHE were purchased from Life Technologies Corporation (USA). 2-(6-Amino-3-imino- 3H-xanthen-9-yl) benzoic acid methyl ester, hydrochloride (Rh123) was purchased from Dojindo Laboratories (Japan). All antibodies used in this study were from Abcam (Abcam, CA, USA). Aconitine, mesaconitine and hyperaconitine were prepared by our laboratory. These compounds were isolated from the ethyl acetate extract of Fuzi lateral root. HPLC-grade acetonitrile (MeCN) were purchased from E. Merck (Darmstadt, Germany) and ammonia (AR grade) was obtained from Beihua Fine Chemicals Co., Ltd. (Beijing, China). The water used for HPLC was purified by a Milli-Q system (Millipore, Milford, MA, USA). Streptozotocin (STZ) was procured from Sigma-Aldrich, USA.

### Induction of Diabetes

Animals were fasted from the evening prior to the day of STZ injection. Streptozotocin (STZ) was dissolved in citrate buffer (pH 4.4) and intraperitoneally injected within 5 min at 65 mg/kg body weight. Age matched control rats received equal volume of vehicle (citrate buffer). 72 h later, tail blood was analyzed using a standard glucometer (One Touch Profile, Lifescan, Inc. Milpitas, CA). Animals showed plasma glucose higher than 16.7 mmol/L were considered as diabetic and were used for diabetic neuropathy studies.

### Treatment Schedule

Diabetic neuropathy was well developed after six weeks of streptozotocin treatment as reported earlier [20]. Treatment with FZE (1.75, 3.50 and 7.00 g/kg body weight, i.g.) was started after the sixth week of diabetes induction. The highest FZE daily dose, 7.00 g/kg body weight, was approximated by the dose given to patients three times per day in Chinese medicine clinic and a higher metabolism of rats, which was equivalent to about 28 times more than a single dose for patients. Motor nerve conduction velocity and hot plate test were measured after two weeks of FZE treatment.

### Motor Nerve Conduction Velocity (MNCV)

Power Lab 8sp instrument (Chengdu Instrument factory, China) was used for the measurement of motor nerve conduction velocity. Briefly, the animals were anesthetized by 50 mg sodium pentobarbital/kg body weight. Motor nerve conduction velocity was measured by stimulating the sciatic (proximal to sciatic notch) nerve with 2 volt, 5 stimulus. The recording electrodes were inserted into skins between toes. Motor nerve conduction velocity was calculated by following formula: MNCV = (distance between sciatic nerve stimulation point and toes skin recording point)/(sciatic M wave latency).

### Hot Plate Test

This test measures the time that elapses before the rat demonstrates hind paw licking/shaking and jumping, which indicate pain in response to the applied heat. The hot plate was maintained at 55±1°C, and the animals were placed into a Perspex cylinder on the heated stage. Response latency was measured by recording the time between placing in the cylinder and shaking or licking the paws. The cut-off time was set at 30 seconds to minimize skin injury.

### Cell Culture and Treatment

RSC96 cells (rat Schwann cell lines) were obtained from Cell Resource Center of Shanghai Institutes for Biological Sciences, Chinese Academy of Sciences. RSC96 Cells were maintained in Dulbecco’s modified Eagle Medium (DMEM) (Hyclone, USA) plus 100 U/ml penicillin, 100 µg/ml streptomycin and 10% (v/v) fetal bovine serum (Gibco, UK) at 37°C in 5% CO_2_ humidified atmosphere.

Cell treatment with FZE was conducted as follows: normal glucose (NG, 5.5 mM glucose), mannitol as osmotic controls(NG+M, 44.5 mM of mannitol plus 5.5 mM glucose), high glucose (HG, 50 mM glucose), 50 mM glucose plus FZE with different concentrations (0.1, 1.0, 10.0 µg/ml) simultaneously. In addition, the cells were treated for 48 h.

### Detection of Intracellular ROS

The contents of intracellular ROS were determined by laser scanning confocal microscope analysis with peroxide-sensitive fluorescent probe DCFH_2_DA and DHE respectively. DCFH_2_DA is mainly oxidized by hydrogen peroxides, while DHE is mainly oxidized by superoxide anions. DCFH_2_DA and DHE are the practice probes used to detect cellular ROS levels in viable cells. In brief, the cells at 1×10^5^/ml in different culture conditions were harvested and treated with 10 µM of DCFH_2_DA or 10 µM of DHE at 37°C for 30 min in the dark. After being washed twice with PBS, the fluorescent intensity of different groups of cells was analyzed by FV1000 (excitation 488 nm for DHE and DCFH_2_DA).

### Measurement of Mitochondrial Membrane Potential (MMP)

RSC96 cells in different culture conditions were harvested and the MMP of those cells were measured with Rh123 assay. In brief, the harvested cells at 1×10^5^/ml were stained with 10 µM Rh123 and incubated at 37°C for 30 min in the dark. After being washed twice with PBS, the fluorescent intensity of different groups of cells was analyzed by FV1000excitation 488 nm.

### Determination of Apoptotic Cells

RSC96 cells were seeded onto 6-well plates at a density of 1×10^6^ cells/well. The cells were starved overnight and treated in triplicate with different concentrations of glucose, as described above. The cells were digested with trypsine, centrifuged and washed twice with PBS. The cells were then incubated in annexin V-PE/7-AAD for 15 min in dark. Stained cells were analyzed using BD FACS Canto II (BD Biosciences). Results from 10,000 events were analyzed in each sample and corrected for auto-fluorescence from unlabeled cells.

### Immunofluorescence

The cells were fixed with 4% paraformaldehyde for 15 minutes at 20°C, permeated with 0.3% triton prior to being blocked in 1% BSA+2% normal goat serum for 30 min at 20°C. Samples were then incubated with primary antibody overnight at 4°C in PBS containing. A FITC goat anti-rabbit polyclonal antibody (ab96899) diluted at 1∶200 was used as the secondary antibody. Cell nucleus were counterstained with DAPI and showed blue. Mitochondria were labeled by Mito tracker (Life Technology, USA) and showed red.

### Statistical Analysis

We applied the Shapiro-Wilk test to verify the normality of the distributions. A two-way analysis of variance (ANOVA) was used to verify the differences between the normal distributions, and the Kruskal-Wallis test was used to assess differences between nonparametric distributions. For normal distributions, the results were expressed as the means ± S.D., and the differences were considered significant when the probability of a Type I error was lower than 5% (p<0.05). For nonparametric distributions, data were expressed as the median, and differences were considered significant when the probability of a Type I error was lower than 0.1% (p<0.001).

## Results

### The MS Analysis of FZE

**Figure 1:**
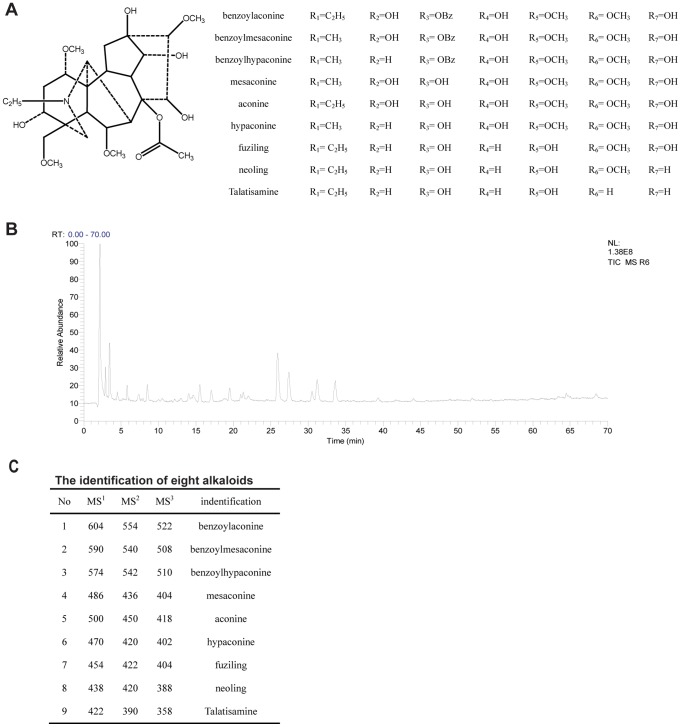
Identification of nine alkaloids. (A) Core stucture and list of corresponding substituents of nine alkaloids. (B) Total ion chromatography of FZE. (C) m/z of nine alkaloids fragmentation ions.

Toxic compounds of Fuzi are diester-type alkaloids. In Chinese medicine, there are many ways to decrease toxicity. The traditional way of preparation (Chinese: Paozhi) or preparation of Fuzi is to boil it by water. In this study, mouse acute toxicity test indicates that intragastrical administration of FZE (64 g/kg), which is up to 256 fold of patient’s daily dose, showed no toxicity. At the same time, when FZE was detected using HPLC-MS^n^ under positive ion mode, there was no diester-type alkaloids found, which are the main toxic ingredients of Fuzi. Nine alkaloids were identified, and the protonated molecular ions of these alkaloids were at m/z 604, 590, 574, 486, 500, 470, 454, 438 and 422. Moreover, all the MS^n^ spectra of these alkaloids displayed a characteristic behavior of loss of CH_3_COOH (60 u), CH_3_OH (32 u), CO (28 u) and H_2_O (18 u). These compounds were characterized as benzoylaconine, benzoylmesaconine, benzoylhypaconine, mesaconine, hypaconine, fuziling, neoling, aconine and talatisamine, separately [18]–[19], [21]–[22]. The results of MS^2–3^ were shown in Figure 1.

### Glucose Level and Body Weight

**Figure 2:**
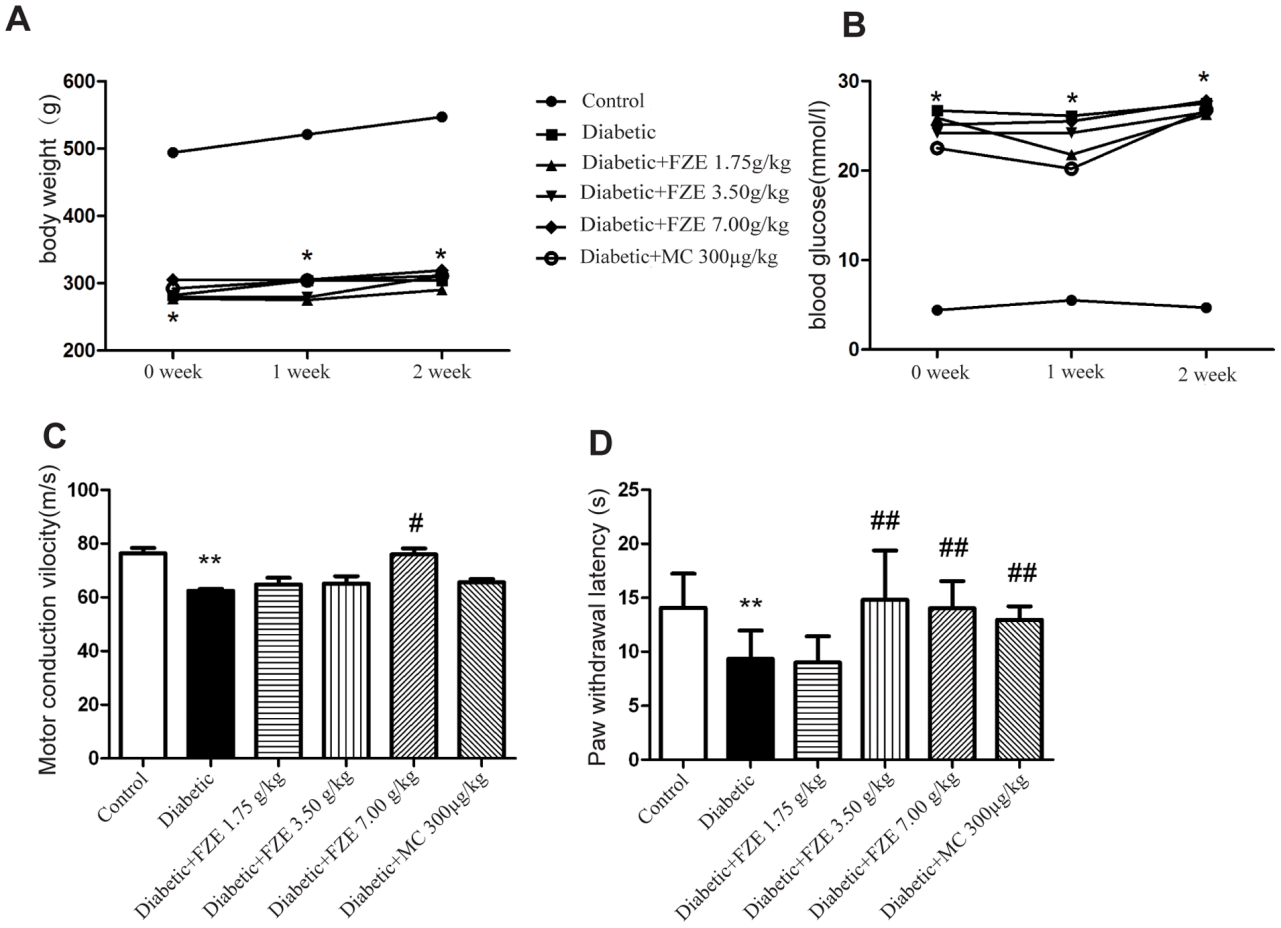
Effects of two weeks treatment of FZE on diabetic rats. (A–B) Effects of FZE on body weight and blood glucose level. Data are expressed as median (n = 6). *: P<0.001, diabetic vs control. (C–D) Effects of FZE on motor nerve conduction velocity and paw withdrawal latency. Data are shown as mean±S.D. (n = 6). **: P<0.01, diabetic vs control. #: P<0.05, ##: P<0.01, compared with diabetic.

After streptozotocin administration, the animals showed 5–6 fold increase in plasma glucose level compared to the age matched control rats (vehicle treated) (P<0.001, Non-parametric test). Treatment with FZE (1.75, 3.50, 7.00 g/kg) did not show significant effect in decreasing blood glucose levels. Besides, slower body weight gain was also observed in the diabetic animals, which was not ameliorated by any of the treatment (Figure 2A–2B).

### Motor Nerve Conduction Velocity

Motor nerve conduction velocity reduced significantly in eight week diabetic rats when compared to age matched control rats. FZE (7.00 g/kg) treatment showed significant reversal (P<0.05, ANOVA test), while other doses showed little effect on motor nerve conduction velocity (Figure 2C).

### Hot Plate Test

When subjected to the hot plate test, diabetic rats exhibited a significant reduction in withdrawal latency as compared to the control rats. Treatment with FZE (3.50 and 7.00 g/kg) and methylcobalamin (300 µg/kg) could reverse the thermal hypoalgesia remarkably (P<0.01, ANOVA test), while FZE (1.75 g/kg) showed no significant protective effect (Figure 2D).

### Effects of FZE on ROS in RSC96 Cells in Response to High Glucose

In this study, 50 mM glucose was added to culture media to set up high glucose environment for RSC96 cells, which was higher than the human serum glucose concentrations. The reason why such a harsh condition was used to lead to SCs injury (in vitro) could due to the immortality of RSC96 cells.

**Figure 3:**
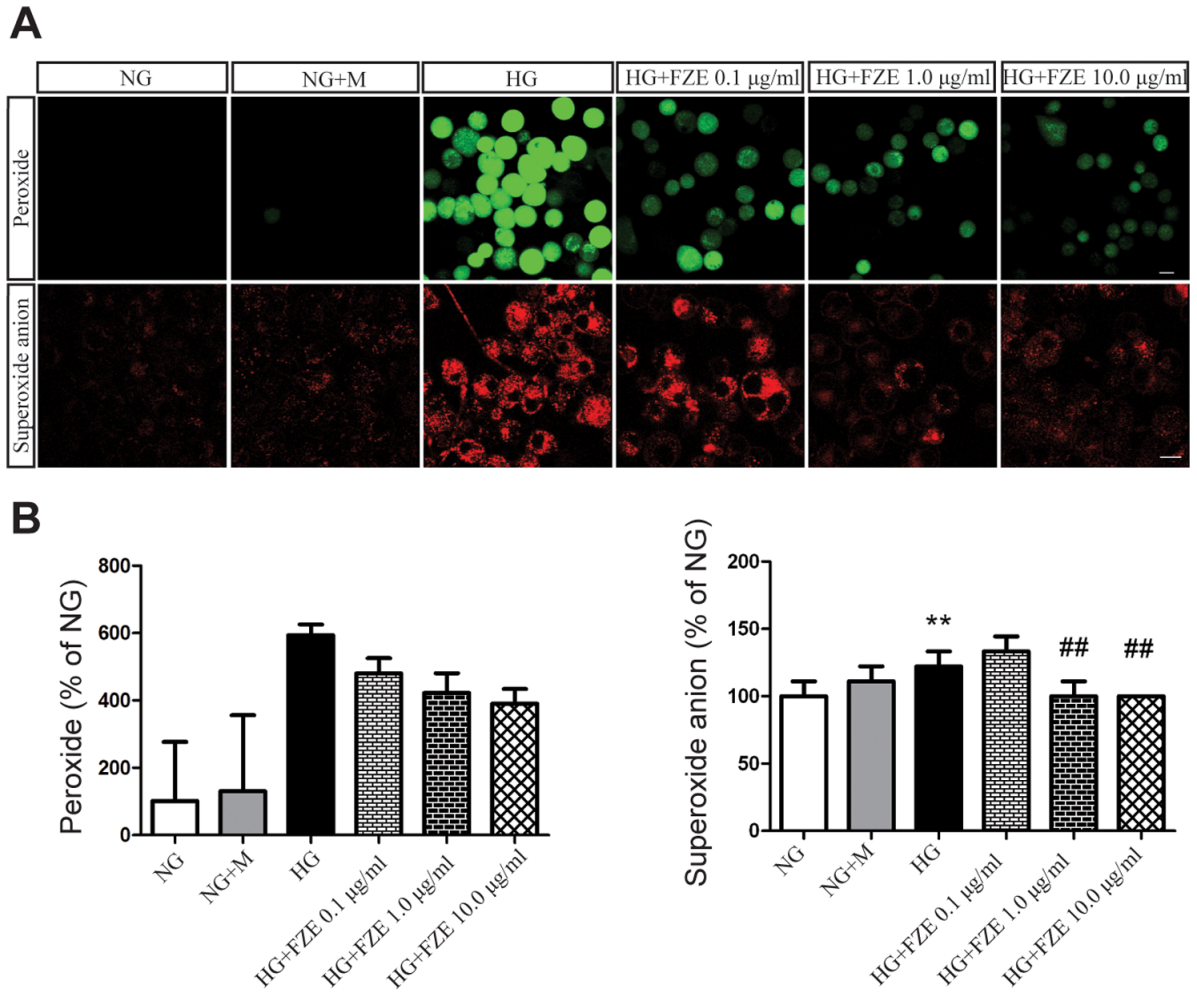
Effects of FZE on ROS levels. (A) Effects of FZE on peroxide and superoxide anion levels. RSC96 cells were stained of DCFH_2_DA and DHE, a fluorescent marker for the peroxide and superoxide anion. (B)Data analysis. Data were represented as mean ± S.D. (n = 3–4). **: P<0.01, HG vs NG, ##: P<0.01, compared to HG. NG (Normal glucose), NG+M (Normal glucose+mannitol), HG (high glucose), HG+FZE (High glucose+Fuzi extract). The ratio is defined as percentage of NG group (being as 100%).

In order to confirm the effects of FZE on the ROS, RSC96 cells were first cultured in media containing 5.5 mM glucose (NG), and then stimulated with NG plus 44.5 mM mannitol (NG+M) or 50 mM glucose (HG) or 50 mM glucose plus FZE with different concentrations for 48 h. The peroxide and superoxide anion were detected using DCFH_2_DA and DHE analysis. We found that the level of peroxide increased within 48 h of HG stimulation (Figure 3), while the increase was not significant as mannitol was added to the NG medium. The addition of FZE to the high glucose medium reduced the intensity of fluorescence stained by DCFH_2_DA which reflect the peroxide level. Incubation with even 0.1 µg/ml FZE could reduce the peroxide level, and the effect was more significant as the concentration rose to 10 µg/ml.

As shown in Figure 3, increased superoxide anion was confirmed under high glucose conditions **(P<0.01, ANOVA test)**. In addition, no differences were found between RSC96 cells cultured under conditions of NG and NG plus mannitol. These findings reveal that ROS were involved in HG-induced injury in RSC96 cells. We found both 1.0 and 10.0 µg/ml FZE could down-regulate superoxide anion level (P<0.01, ANOVA test).

### Effects of FZE on MMP in RSC96 Cells in Response to High Glucose

**Figure 4:**
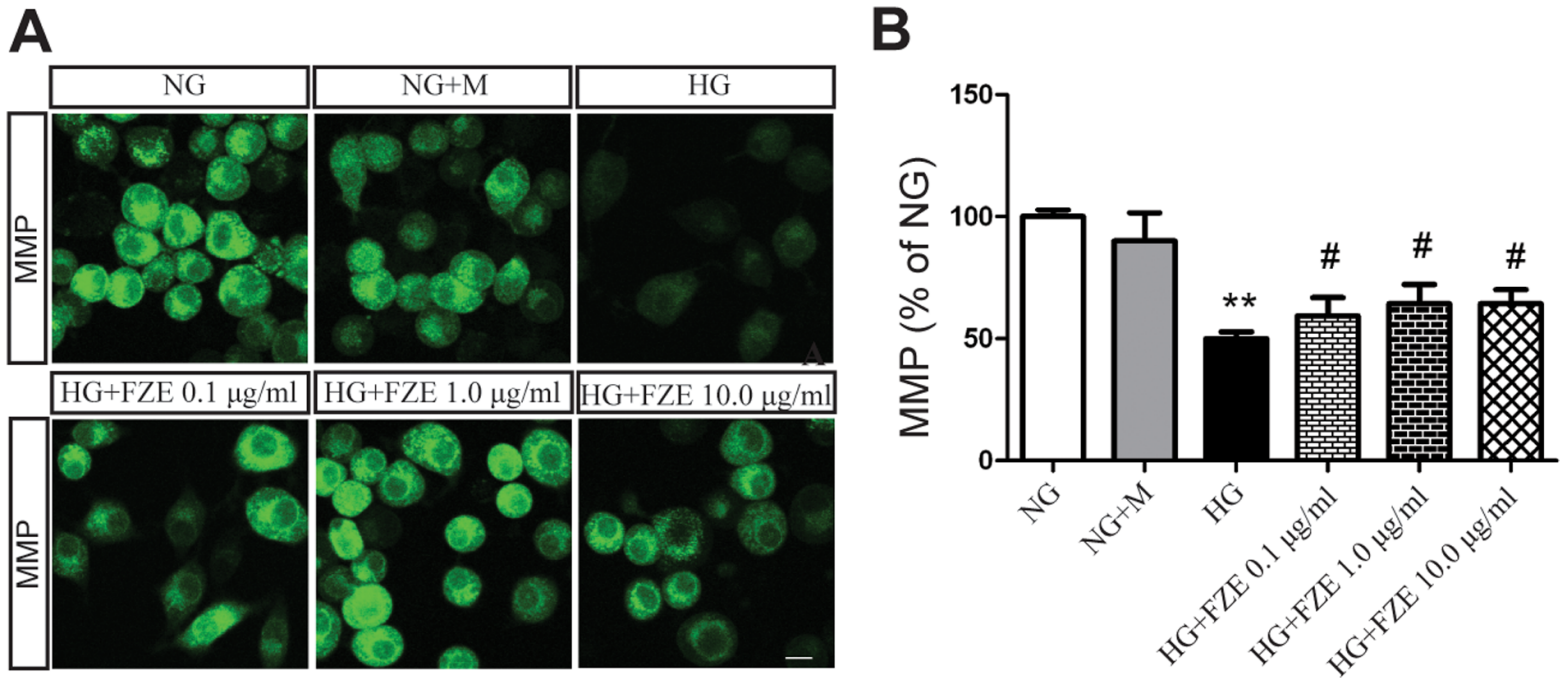
Effects of FZE on level of MMP in RSC96 cells. Cells were stained of Rh123, a fluorescent marker for MMP. Data were represented as mean ± S.D. (n = 3). **: P<0.01, HG vs NG; #: P<0.05, compared to HG. NG (Normal glucose), NG+M (Normal glucose+mannitol), HG (high glucose), HG+FZE (High glucose+Fuzi extract). The ratio is defined as percentage of NG (being as 100%).

The fluorescence of Rhodamine 123 staining was used to measure the MMP which drives the uptake and accumulation of Rh123 in the mitochondria. The hypofluorescence peak observed was indicative of a collapse in the MMP and depolarization of the mitochondrial membrane. As shown in Figure 4, significant reduction in the fluorescent intensity and a collapse of the MMP were observed in the RSC96 cells cultured with high glucose for 48 h (P<0.01, ANOVA test). After treatment with various concentrations of FZE, the fluorescent intensity of RSC96 cells were recovered, indicating the depolarization of the mitochondrial membrane was restored, and 1.0–10.0 µg/ml FZE gave the best effect (P<0.05, ANOVA test).

### Effects of FZE on Apoptosis of RSC96 Cells in Response to High Glucose

**Figure 5:**
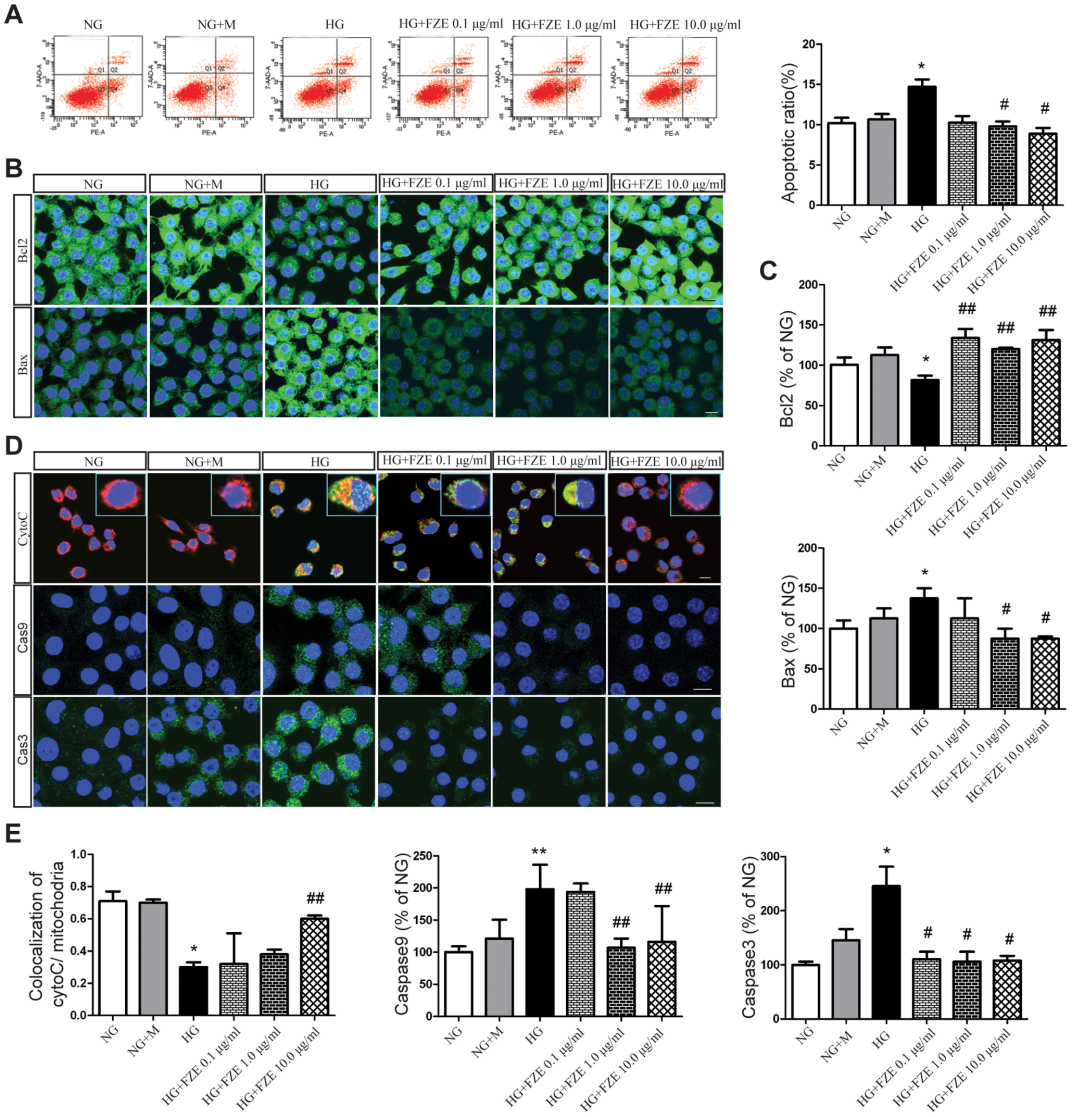
Effects of FZE on apoptotic ratio and apoptotic factors in RSC96 cells. (A) Effects of FZE on apoptotic ratio. Apoptosis was assessed using flow cytometry with annexin v-PE/7-AAD staining. (B–C) Effects of FZE on Bcl2 and Bax levels. The Bcl2 and Bax were examined using immunofluorescence and FITC labeled secondary antibody was used. Nucleus was label by DAPI. (D–E) Effects of FZE on translocation of CytoC andthe levels of caspase9 and caspase3. The mitochondria were labeled by Mito tracker; the levels of CytoC, caspase9 and caspase3 were examined using immunofluorescence and FITC labeled secondary antibody was used. Nucleus was label by DAPI. Data were represented as mean ± S.D. (n = 3–4). *: P<0.05, **: P<0.01, HG vs NG; #: P<0.05, ##: P<0.01, compared to HG. NG (Normal glucose), NG+M (Normal glucose+mannitol), HG (high glucose), HG+FZE (High glucose+Fuzi extract). The ratio is defined as percentage of NG (being as 100%).

To verify the antiapoptotic effect of FZE on RSC96 cells, flow cytometric analysis was conducted using dual staining with annexin V-PE and 7-AAD, which were used to distinguish viable, early apoptotic, late apoptotic or necrotic cells. As shown in figure 5A, there was no apparent apoptotic ratio change between mannitol (M) and normal glucose (NG) group cells. As expected, the apoptotic cell ratio increased markedly in RSC96 cells stimulated with high glucose (HG, 50 mM glucose) compare to NG group (P<0.05, ANOVA test). Treatment with both 1.0 and10.0 µg/ml FZE obviously decreased the apoptotic ratio in comparison with HG group (P<0.05, ANOVA test, Figure 5A), and the effect was more significant as the concentration rose to 10.0 µg/ml.

### Effects of FZE on Bax and Bcl2 in RSC96 Cells in Response to High Glucose

To determine whether Bax and Bcl2 plays a role in FZE mediated antiapoptotic effect, we assessed the Bax and Bcl2 protein level of RSC96 cells after FZE treatment using Immunofluorescence (Figure 5B). Treatment of cells for this test included normal glucose, normal glucose plus mannitol, high glucose, and high glucose with the addition of 0.1–10.0 µg/ml FZE. Cells were treated for 48 h. After fixation, Bax and Bcl2 level were examined by measuring fluorescence intensity. The Bax level cultured in normal glucose was similar to that with mannitol treatment. With high glucose treatment (50 mM), the Bax level was up-regulated (P<0.05, ANOVA test, Figure 5C). FZE with concentration of 1.0–10.0 µg/ml showed significant effects in reducing bax levels (P<0.05, ANOVA test, Figure 5C), while 0.1 µg/ml FZE showed mild effect. Compared to cells in normal glucose, there were significant changes in Bcl2 level in high glucose treated cells (P<0.05, ANOVA test, Figure 5C), but not in cells stimulated with mannitol. Bcl2 level was significantly lower in FZE treated cells versus high glucose only cells (P<0.01, ANOVA test, Figure 5C). No obvious difference was found among different concentrations of FZE.

### Effects of FZE on Translocation of CytoC from Mitochondria in RSC96 Cells in Response to High Glucose

To determine whether FZE suppressed translocation of CytoC from mitochondria, we examined the colocalization of CytoC and mitochondria. In NG group, most CytoC existed in the mitochondria, with little or no CytoC located in the cytosol (Figure 5D). Translocation of CytoC was significantly increased in the HG group compared to the NG group (P<0.05, ANOVA test, Figure 5E), suggesting that mitochondrial permeabilization was induced after high glucose stimulation (P<0.05, Figure 5E). FZE significantly attenuated mitochondrial CytoC translocation remarkably compared to HG group cells. These differences became significant when FZE rose to 10.0 µg/ml (P<0.01, ANOVA test, Figure 5E).

### Effects of FZE on Caspase9 and Caspase3 in RSC96 Cells in Response to High Glucose

To investigate how FZE inhibited apoptosis in RSC96 cells, we examined the expression level of apoptosis-related proteins, including cleaved-caspase3 and cleaved-caspase9 which are generally activated by the mitochondria-dependent apoptotic pathways. As shown in Figure 5D, in NG plus M group, the level of caspase-3 also increased slightly in RSC96 cells, which could due to higher osmolality. After exposure to high glucose for 48 h, the cleaved-caspase3 and cleaved-caspase9 level was significantly elevated (P<0.05 or P<0.01, ANOVA test, Figure 5D–5E). We observed that the expression of cleaved-caspase3 and cleaved-caspase9 proteins was decreased significantly with FZE treatment (P<0.05 or P<0.01, ANOVA test, Figure 5D–5E).

## Discussion

The only “proven” therapy for reducing risk and slowing progression of diabetic neuropathy is aggressive glycemic control. No treatment has proven effective at preventing or slowing the progression of diabetic neuropathy, although a number of therapies in cell culture and animal models of diabetic neuropathy are promising [23]. Moreover, some therapies are with serious side effect, which impair life quality of patients.

Fuzi is one of the most popular herbs for pain and paralysis (Chinese: Weizheng). In traditional Chinese medicine, paralysis has been described as fatigability, cold in the extremities, leg numbness and pain [24], [9]. Fuzi is considered to be a useful approach for the improvement of subjective symptoms such as numbness, sensation of cold and pain in the extremities [9], [10], [12], [24] which are associated with diabetic neuropathy. One of the noteworthy characteristics of Fuzi is that its application has been passed on for thousands of years. The current doubt over the role of Fuzi in cure of diabetic neuropathy revolves around its ability to serve as an independent monotherapy and its toxicity.

In this paper, FZE was used to assess the anti-diabetic neuropathy effect of Fuzi both in animal model and in RSC96 cells. Nine alkaloids, which are superior to diester-alkaloids (the most toxic components in Fuzi) in toxicity and stability, were identified in FZE. To ensure the reproducibility of the identification, peak areas of these nine compounds from 6 parallel experiments were detected. The precision were 1.26% (m/z 604), 1.36% (m/z 590), 1.32% (m/z 574), 2.15% (m/z 500), 2.08% (m/z 486), 2.98% (m/z 470), 3.25% (m/z 422), 3.18% (m/z 438) and 2.95% (m/z 454), respectively. So, the preparation technology of FZE is stable, simple, and controlled, which is suitable for industrialization. Besides, the safety of FZE was also proved by acute toxicity test.

In animal experiment, after eight weeks of diabetes induction, we observed significant reduction in MNCV with decreased paw withdrawal latency for hot plate tests. These results indicate development of diabetic neuropathy and are consistent with previous reports [20]. Treatment with FZE at 7.00 g/kg, which started after the sixth week of diabetes induction and lasted for two weeks, could significantly improve the nerve conduction deficits and thermal hypoalgesia deficits in the diabetic rats. We also note that the levels of blood glucose in FZE groups showed little reduction, which indicates that the protective effect of Fuzi on diabetic neuropathy was independent of dramatic interference with glucose level.

Methylcobalamin, which is one of the co-enzyme forms of vitamin B12 and acts as an important co-factor in the activities of vitamin B12-dependent methyltransferases [25]–[26], is usually used to ameliorate diabetic neuropathy both in clinic and in experimental study. In our study, methylcobalamin (300 µg/kg, treatment for 2 weeks) could effectively improve paw withdrawal latency, but showed little effect on nerve conduction velocity. Previously, it was reported that a significant increase of motor nerve conduction velocity was shown in the methylcobalamin (500 µg/kg or 300 µg/kg) treated diabetic rats over 8 or 16 weeks treatment [27]–[28]. It suggests that poor effect of methylcobalamin in our study might be attributed to the shorter duration or lower dose. Furthermore, it is also concluded that the onset time of Fuzi effect is earlier than methylcobalamin.

Schwann cells generate and maintain a multi-lamellar insulating myelin sheath around an associated axon and impose cellular specializations that allow fast conduction of action potentials [29]. Reduced MNCV in the diabetic peripheral neuropathy is principally due to the impaired function of SCs [30]. Given the important role of SCs in supporting neuronal function, they may serve as the target of Fuzi treatment of diabetic neuropathy.

ROS has been proposed as a possible mechanism for high glucose-induced Schwann cell dysfunction in both in vitro and in vivo studies, which is involved in the pathology of DPN [31]–[32]. Mitochondria are both important sources and targets of ROS, and are also the essential organelle involved in cell apoptosis. Mitochondrial membrane potential is a sensitive indicator reflecting the mitochondrial function. The decline of mitochondrial membrane potential was correlated with opening of permeability transition pore, which leads to the release of apoptosis-activating proteins [33]. Pro-apoptotic Bax and anti-apoptotic Bcl-2 family proteins on the mitochondrial outer membrane are believed to play an important role in cell survival. With apoptotic stimuli, Bax is post-transcriptionally activated, oligomerized and translocated to mitochondria, and then it triggers CytoC releasing from mitochondria. CytoC binds to apoptosis protease activating factor-1 (Apaf-1) and leads to the assembly of an apoptosome complex. This apoptosome can bind procaspase-9 and cause its auto-activation through a conformational change. Once initiated, caspase-9 goes on to activate caspase-3 (effector caspase), which cleaves substrates at aspartate residues, such as caspases-6 and −7. They are executioner caspases and activate a DNase which is responsible for the fragmentation of oligonucleosomal DNA [34]. Apoptosis has also been associated with diabetic neuropathy [35]–[36].

In the present study, high glucose in RSC96 cells induced a significantly high level of ROS, promoted significant reduction in the MMP, and produced an increase in apoptosis compared to NG cells, which is in agreement with previous studies. We further investigated the effect of FZE on High glucose induced detrimental effect, and found that FZE could effectively decrease ROS, improve MMP elicited by high glucose at a dosage dependent manner. It is also noticeable that the apoptotic ratio decrease from14.70% in HG cells to 10.27% in NG cells (43% of NG).

Furthermore, our study found that FZE downregulated high glucose-induced elevation of proapoptotic protein Bax and upregulated Bcl-2 expression, which was accompanied by decreased translocation of CytoC, downregulated active caspase-9 and reduced active caspase-3 activation. These results demonstrate that the antiapoptotic effect of Fuzi was probably due to mitigated glucose-induced mitochondrial dysfunction.

The present study was a comprehensive evaluation of Fuzi, combining with phytochemical, physiological and electrophysiological studies. In conclusion, Fuzi ameliorate diabetic neuropathy via inhibiting Schwann cells apoptosis which is mediated by mitochondrial pathway.
